# Three-Dimensional Finite Element Analysis of the Undercut Anchor Group Effect in Rock Cone Failure

**DOI:** 10.3390/ma13061332

**Published:** 2020-03-15

**Authors:** Józef Jonak, Michał Siegmund, Robert Karpiński, Andrzej Wójcik

**Affiliations:** 1 Department of Machine Design and Mechatronics, Faculty of Mechanical Engineering, Lublin University of Technology, Nadbystrzycka 36, 20-618 Lublin, Poland; r.karpinski@pollub.pl (R.K.); a.wojcik@pollub.pl (A.W.); 2 KOMAG Institute of Mining Technology, Pszczyńska 37, 44-100 Gliwice, Poland; msiegmund@komag.eu

**Keywords:** FEM, fracture mechanics, numerical modeling of fracture, rock mechanics

## Abstract

An objective of this study was to investigate the group effect in rock cone failure occurring in pull-out with the use of 3D finite element analysis. At present, undercut anchors are typically applied as structural fasteners of steel elements in concrete buildings; however, new areas for their use are being explored. The reported study set out to evaluate the use of undercut anchors in special-purpose rock mining, e.g., in mining rescue operations. In such emergencies, mechanical mining may prove impossible, whereas the use of explosives is even prohibited. Although manual methods could be considered, their effectiveness is hard to assess. Prior to considering the use of undercut anchors in mining, several aspects must essentially be determined: The mechanics of cone failure, including the extent of surface failure and the values of the pull-out force of the anchor for a given rock mass relative to the anchor system, the embedment depth, or the rock strength parameters. These factors may be investigated successfully using finite element analysis, the results of which are presented in the study.

## 1. Introduction

Undercut anchors are primarily considered in use as fasteners in steel elements of concrete structures. The design of fastenings for use in concrete is currently based on the recommendations given in the European Standard CEN/TS 1992-4 [1].

According to the EU Standard [2], the failure of head anchors occurs as one of the following modes:

- pull-out failure,
- concrete breakout failure,
- concrete blow-out,
- concrete splitting,
- steel failure of fastener.

Each of the failure modes above is characterized by a different value of the load required and the form of failure (specifically, the volume of the removed base material).

The currently accepted empirical models describing the mechanism of the breakout prism formation have been developed from the numerous experimental studies.

Publications [1,2,3,4,5,6,7] contain the main information related to the terminology and basic standards for anchor fixing techniques. The works in this field carried out to date have focused on determining the load-bearing capacity of anchors depending on their construction, physical and mechanical parameters of the medium in which they are to be installed, and the influence of the installation technology [8,9,10,11,12]. Simultaneously, the literature survey indicates that analytical models of crack trajectory propagation and the formation of the breakout cone are predominantly based on experimental data and linear elastic crack mechanics [7,13,14]. In recent years, computational methods and numerical analyses using FEM systems, mainly ABAQUS, have been on the rise. In addition, 2D and 3D models of the interaction of anchors on the medium are being improved; in particular, the optimal spacing of anchors in multi-anchor systems is being sought [3,5,6,12,15,16].

The two most notable models are the traditional 45° cone approach (ACI Method 349-85) and the concrete capacity design (CCD) method [5], where the angle of the failure surface (cone or pyramid) is 35° [6]. The damage in the form of a concrete cone is idealized as a pyramid with a height equal to the effective embedment depth—*h*_ef_. In principle, the base of the designed concrete failure cone for a single anchor is approximately a square with a side of ~3*h*_ef_ [4].

These models provide the basis for estimating the load capacity of anchors and the approximate extent of failure on the free surface of the base material. The general load capacity of anchor fastenings depends on the minimum pull-out force applied under the given process conditions.

In the 45° cone method (ACI 349-90 [1]), the load capacity of the anchor is determined from the relationship below:

(1) $$N_{u,m}=0.96\pi f_{cc}^{0.5}h_{ef}^{2}\left(1+\frac{d_{h}}{h_{ef}}\right),$$

where:

*N_u,m_*—mean anchor load capacity (N),
*f_cc_*—compressive strength, cubic sample (N/mm^2^),
*h_ef_*—effective embedment depth, (mm),
*d_h_*—mean anchor head diameter, (mm),
0.96 (N^0.5^/mm^0.5^)—calibration factor.

In practice, however, the load-carrying capacity of undercut anchors is currently determined from the empirical relationship, rooted in the procedures based on experience and tests (including CCD) [1,7]:

(2) $$N_{u,m}=k_{1}f_{c}^{0.5}h_{ef\;}^{1.5},$$

where:

*N_u,m_*—mean pull-out load (load capacity of anchors) (N),
*k*_1_ (N^0.5^/mm^0.5^)—calibration factor, accounting for, e.g., units in the model, anchor type, base material, embedment depth, etc.,
*f*_c_—compressive cylinder strength of concrete (N/mm^2^),
*h_ef_*—effective embedment depth (mm).

The 45° cone method tends to vastly overestimate the hypothetical anchor load capacity in comparison to the values obtained in practice. Therefore, as it exhibits a closer fit with the experimental results, it is the CCD method that is a recommended reference.

Due to further discrepancies between the theoretical and actual failure modes with respect to the elastic linear fracture mechanics and non-linear fracture mechanics, a number of models have been proposed for the estimation of the load capacity of anchors (the value of the pull-out force) and the extent of failure on the free surface of a base material (e.g., Eligehausen [13], Piccinin [14], Brincker [8]).

Studies have shown that the depth of anchor deposition, commonly referred to as the effective embedment depth, plays an important role in the 2D and 3D models of crack propagation. For small embedment depths, the crack propagation angle (measured relative to the surface normal to the anchor axis) amounts to approx. 28°, whereas at greater depths, it can reach angles of up to 45° [17].

Based on linear elastic fracture mechanics (LEFM), Eligehausen and Savade [13] developed an analytical/theoretical model, which enables the potential anchor load capacity to be determined from the following relationship:

(3) $$F_{EG_{f}}=a_{1}{\left(EG_{f}\right)}^{0.5}h_{ef}^{1.5}f\left(\frac{a}{l_{B}}\right),$$

(4) $$\frac{a}{l_{B}}=0.45,$$

(5) $$F_{EG_{f}}=2.1{\left(EG_{f}\right)}^{0.5}h_{ef}^{1.5},$$

In this model, the load capacity (anchor pull-out strength) *F_EGF_* (N) is a function of the calibration factor *a*_1_, the anchoring depth *h_ef_* (mm) in the exponent 1.5, the modulus of elasticity of concrete *E*, and the fracture energy of concrete *G_F_*. The critical calibration factor *a*_1_ = 2.1 (N^0.5^/mm^0.5^) links the anchor load capacity (peak load) with the length of the propagating crack at loss of stability, compared to its extrapolated total crack length to the concrete surface, while *a* is actual crack size measured along the crack path and *l_B_* is the projected crack path (at an angle of 37.5° to the horizontal direction) in (mm).

Numerous studies focus on the group effect in cone failure [11] that occurs when the critical anchor spacing is not retained (i.e., ~3*h*_ef_), thus causing a reduction in the load capacity of the group of anchors or a decrease in the ultimate pull-out force (e.g., [9]).

The major factor governing the group effect in cone failure is the anchor arrangement and, specifically, the ratio between the anchor spacing (minimum spacing between the anchor axes) to the effective embedment depth [12,18,19].

The average resistance of a system of anchors to tensile loading in unreinforced and non-cracked concrete is calculated from the following relationship [20]:

(6) $$N_{Rm,c}=N_{Rm,c}^{0}\frac{A_{c,N}}{A_{c,n}^{0}}\psi_{s,N}\psi_{eC,N}.$$

where:

$N_{Rm,c}^{0}$—resistance of a single anchor,
$A_{c,n}^{0}$ = 9*h*^2^*_ef_*—projected area of concrete section of a single anchor with edge distance equal to or greater than the minimum allowable distance of 3*h_ef_*,
$A_{c,N}$—projected actual concrete failure area of a group of anchors including the spacing and edge distance,
$\psi_{s,N}$—the modification factor to account for the influence of edges of the concrete member on the stress distribution in concrete,
$\psi_{eC,N}$—the modification factor to account for the group effect when different tension loads are acting on individual anchors of a group.

Notably, 2D and 3D FEM modelling are established methods employed for years in the analysis of base material failure. The major drawback of the computational algorithms (including ABAQUS) lies in their slight inaccuracy, which causes a certain discrepancy of the final phase of the modelled crack propagation from reality (e.g., [16]). The literature provides varying explanations for this, e.g., indicating that it occurs as a result of the change in the equilibrium conditions of the separated mass and the emergence of the bending moment [21].

As a result, compressive stresses appear and the crack begins to propagate almost parallel to the free surface of the medium [22].

According to Maruyama et al. [23], when bending occurs beyond (1.7–1.9)*h*_ef_ from the center of the bolt, it has a marginal impact on the load-carrying capacity of anchors: The crack propagates uncontrollably, almost parallel to the free surface [23].

The proposed mechanism has been confirmed by 3D finite element models (e.g., [10]), which show a similar crack path, including the distinct change in the end-stage. The crack trajectory in its final stage of development occurs in the FEM ABAQUS system. The gap begins to “wrench,” i.e., to penetrate parallel to the free surface, or even penetrate into the material instead of exiting the free surface. This shows that the FEM ABAQUS system algorithm cannot correctly determine the direction of crack propagation in this zone. The crack progresses toward the upper surface but does not reach it.

Notably, 3D FEM systems are highly suitable for the analysis of breakout prism formation (surface delamination) and for modelling how changes in the effective embedment depth or the distance between anchors, in two-anchor and multiple-anchor systems, affect the shape of the breakout cone [15]. This mechanism is essential not only from the perspective of fastening but also from the viewpoint of different applications of breakout technology. Equipped with the theoretical models and data, engineers are able to drill holes for anchors that ensure a maximum breakout prism volume in given geological and technological conditions, which translates to substantial progress in the removal of base material.

## 2. Numerical Experiment—3D FEM Analysis of Crack Propagation

Depending on the circumstances, mechanical methods of extraction may not always be applicable in rock mining. The major restriction for employing mechanical or explosive means for rending rocks, e.g., in rescue operations, is the presence of methane. These exceptional circumstances may necessitate employing manual breakout methods, whose effectiveness is, however, difficult to assess. Considering these difficulties, it appears that concrete breakout with anchors could successfully substitute the aforementioned methods.

Prior to applying the method in field conditions, it is essential to study the breakout mechanism with respect to the breakout cone size, the behavior of the pull-out force for a given anchor assembly in a given rock mass, as well as the group effect, embedment depth, or strength characteristics of the considered base material. The efficiency of drilling can be assessed by the volume of the base material removed by a single anchor or a group of anchors, which is subsequently reflected by the energy consumption of the process or the progress of excavation. The emerging question is whether the efficiency of base material removal with a single anchor is higher than with a group of anchors.

At the stage of the study reported in this paper, the 3D FEM method was employed to estimate the volume of the breakout prism under specific conditions of the anchor–rock interaction. One-, two-, and three-anchor systems were considered.

### 2.1. Cone Failure of a Single-Anchor Fastener

The subject of the study was the Hilti HDA-P undercut anchor [24]. In the simulations, different anchors sizes were modelled; the input data for modelling were derived from the technical specifications given in the Hilti catalogue (M12 and M16 or M20 anchors).

**Figure 1:**
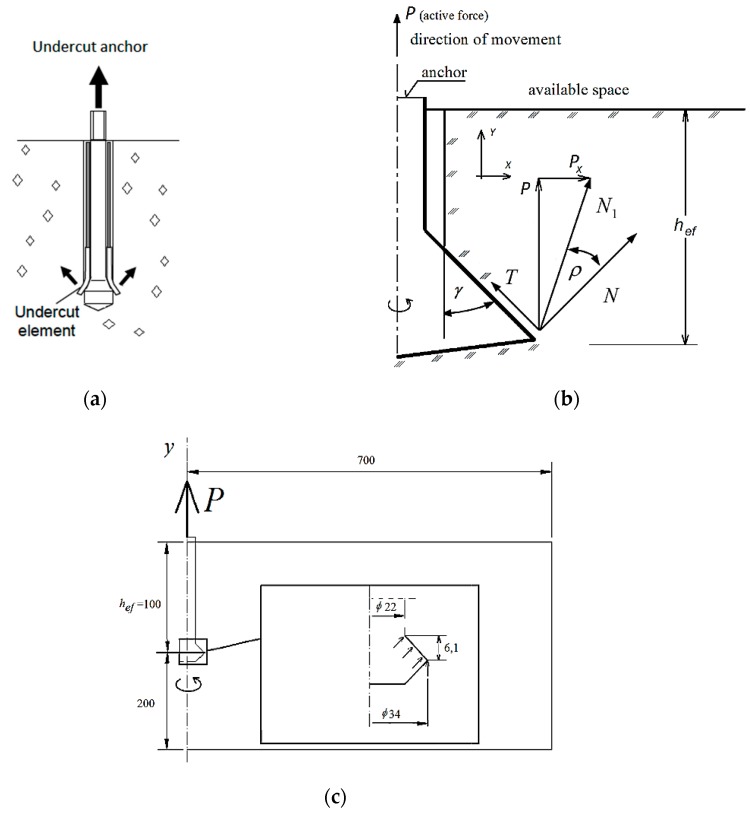
(**a**–**c**) 3D model of a single-anchor fastener.

The numerical analysis was carried out using the eXtended Finite Element Method (XFEM) in ABAQUS (Abaqus 2019, Dassault Systemes Simulia Corporation, Velizy Villacoublay, France). The geometric model is shown in Figure 1. The 3D model was obtained by rotating the 2D anchor model (Figure 1a) around its longitudinal axis. Figure 1b shows a section of the model. A simplified model showing the performance of the headed anchor (the conical part) in the rock material was created by applying elementary force vectors on the nodes of the hypothetical anchor–rock contact surface. The dimensions of the simulated geometrical model were the following: *L* = 700 mm, *h_ef_* = 100 mm (Figure 1c).

Results from the simulations show [3] that the coefficient of friction of the anchor surface against the base material, or, as seen from the model, the breakout cone angle *γ*, affects the crack trajectory when the friction angle *ρ* = 0°. For the coefficient of friction *µ* = 0, the force applied by the anchor on the base material is normal to the element of its cone (then, *µ* = tg*ρ* = 0 ⇔ *T* = 0, *N*_1_ = *N, N* = *P*/cos*γ*).

**Figure 2:**
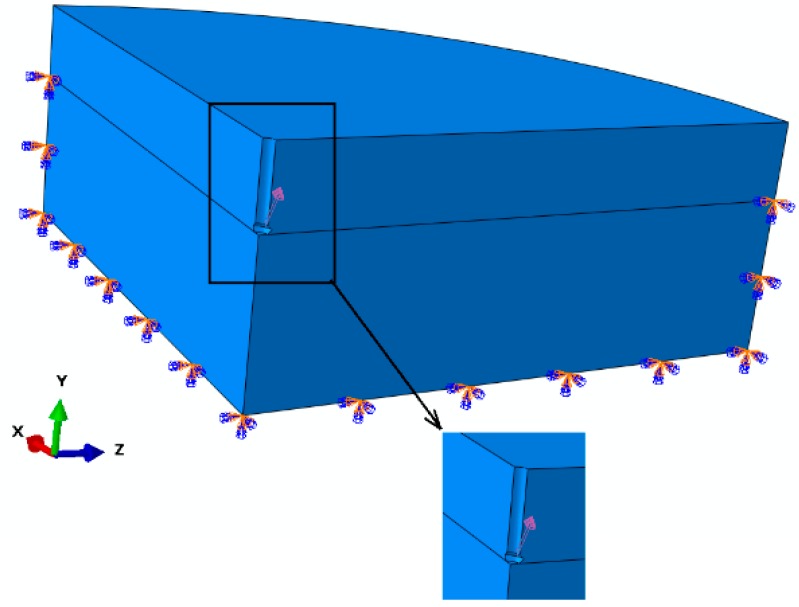
The boundary conditions of the model.

**Figure 3:**
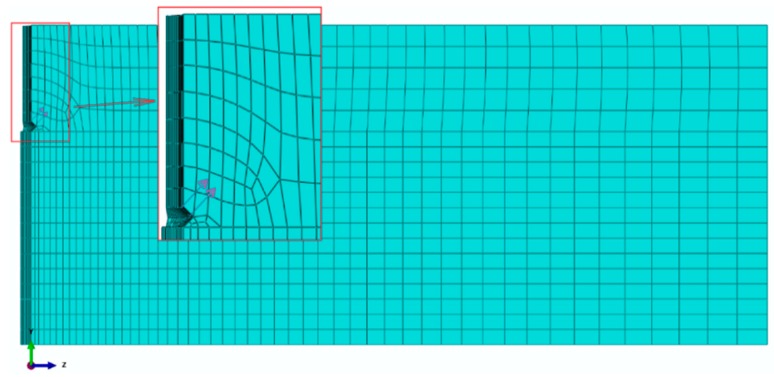
The model discretization method—the modelled quadrant.

The active force (pull-out force) *P*, working along the OY axis [3] (Figure 1b), is substituted by an equivalent load assigned to the nodes of the hypothetical anchor–rock contact surface, as in Figure 2 and Figure 3. For the coefficient of friction *µ* = 0, *P = ∑N_i_* cos*γ*. The factor *γ* is characteristic of a given anchor head after undercutting and expansion in the base material. An M12 anchor and its characteristic parameters are given in [3] Figure 1c.

The modelled base material was sandstone, described by the following parameters: Linear elastic, Young’s modulus *E* = 14,276 MPa, Poisson’s number *ν* = 0.247, tensile strength *f_t_* = 7.74 MPa, and energy of fracture *G_fc_* = 0.335 N/mm.

The following constraints were applied: Outer vertical face, lower lateral and bottom face—restrained, central vertical planes—symmetry (Figure 2).

The discretization of the model (finite element mesh) is illustrated in Figure 3.

Details of numerical analyses of the impact of a single anchor and the results obtained are presented in other papers [3,5,16,25].

### 2.2. Cone Failure of the Group of Two Anchors

**Figure 4:**
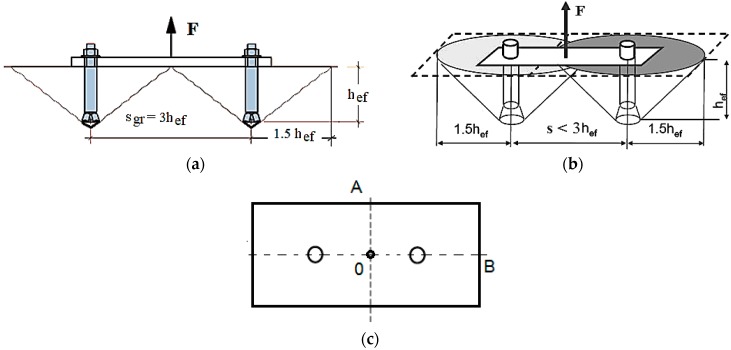
A scheme of a two-anchor system of fasteners: (**a**) the limit distance of the anchors, (**b**) the interaction of the failure cones, (**c**) the impact model quarter of 2 anchors analyzed.

**Figure 5:**
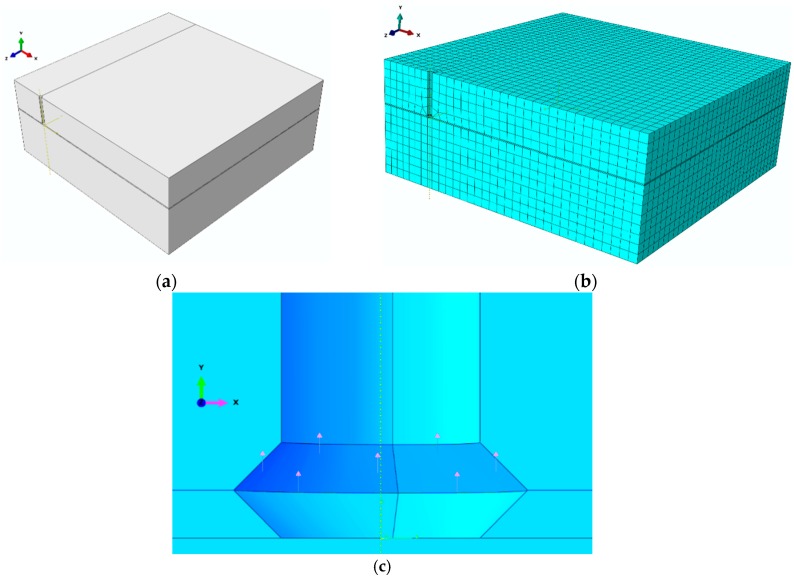
(**a**) A quadrant model for two anchors, (**b**) FEM mesh discretization, and (**c**) equivalent load for the interaction of the anchor cone on the base material.

The investigation of the group effect in cone failure occurring in a system of 2 anchors (Figure 4) was based on the model shown in Figure 5.

Figure 4 shows that in group 2 of the anchors the load F was distributed through the traverse so that the tensile forces in each anchor were equal.

Due to the axial symmetry, Figure 5a shows a quadrant of the 3D FEM model of a group of two M12 anchors, with an A0B cross-section, as in Figure 4. For the considered quadrant, the anchor was modelled to move along the OY axis of the coordinate system (Figure 5). The tensile force was replaced with the load distributed in mesh nodes in the anchor cone (as in Figure 5c).

The following restraints were defined: Outer vertical and bottom face—restrained, central vertical planes—symmetry, as well as energy damage evolution and linear softening.

**Model and material parameters:**

*E* = 14.276 MPa—Young’s modulus,
*ν* = 0.247—Poisson’s ratio,
Critical principal stress *f_t_* = 7.74 MPa—tensile strength,
Damage evolution: Softening linear,
Critical fracture energy rate: *G_fc_* = 0.335 N/mm,
Damage stabilization: Cohesive with viscosity coefficient = 1 × 10^−6^.

**Mesh parameters:**

ABAQUS element: C3D8R—8-node linear brick with reduced integration,
Total number of nodes: 26,517,
Total number of elements: 23,694.

The geometric models differed in the spacing between the holes: a) 100, b) 150, c) 200, and d) 300 mm, with constant effective embedment depth *h_ef_* = 100 mm. In the subsequent part of the analysis, *h_ef_* = 50 mm and the anchor spacing *s* was: e) 50 mm, f) 100 mm.

**Figure 6:**
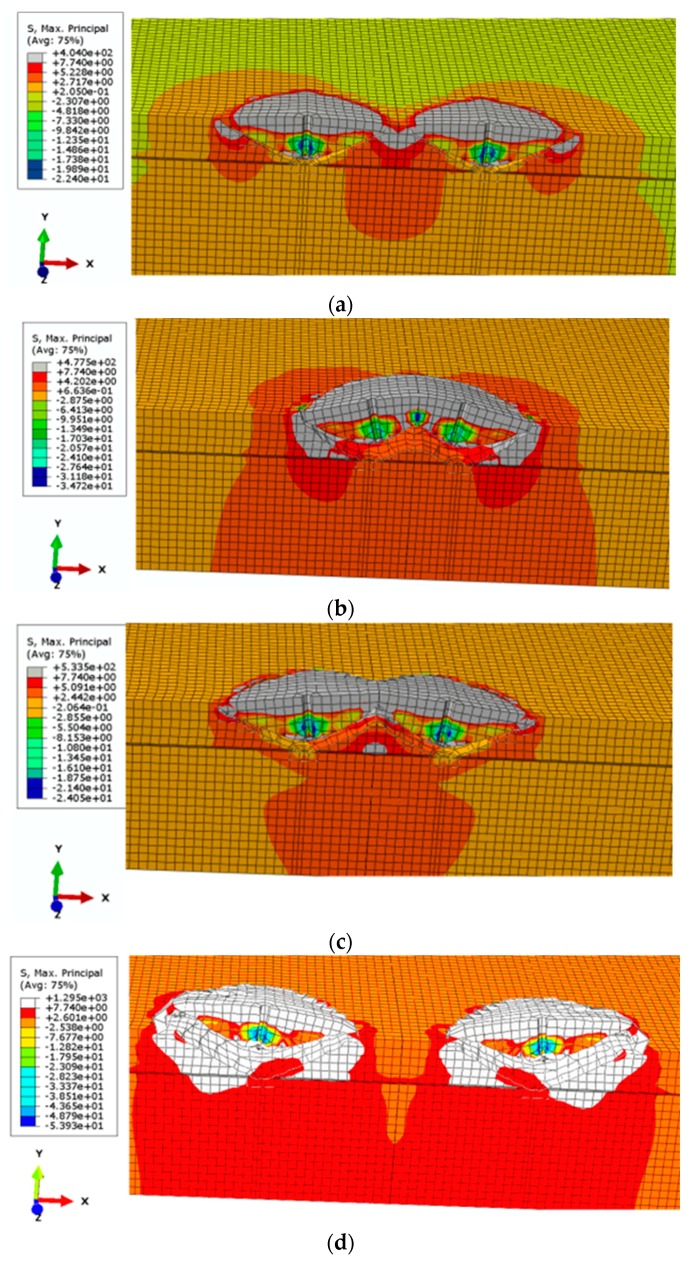

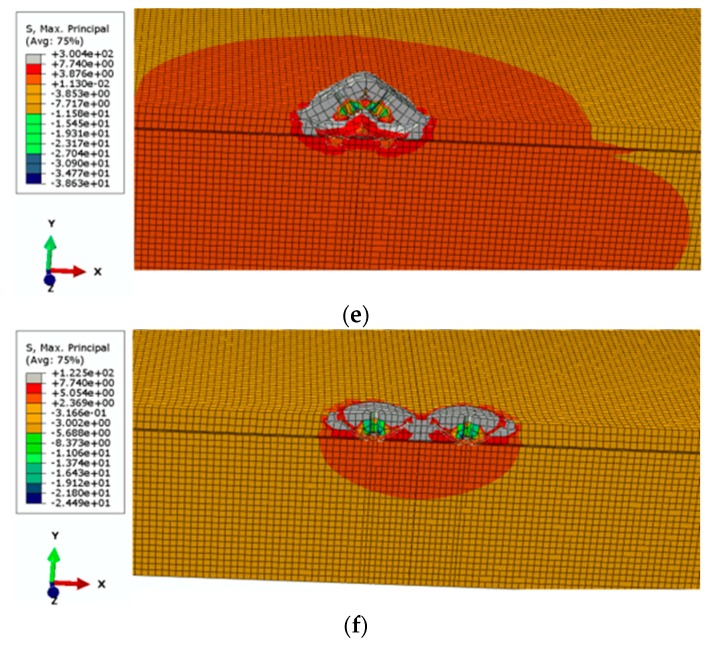
The anchor group effect in cone failure for a group of 2 anchors relative to anchor spacing: (**a**–**d**) *h*_ef_ = 100 mm and the spacing *s* between the holes: (**a**) 100, (**b**) 150, (**c**) 200, **d**) 300 mm, (**e,f**) *h*_ef_ = 50 mm and the spacing *s* between the holes: (**e**) 50 mm, (**f**) 100 mm.

**Figure 7:**
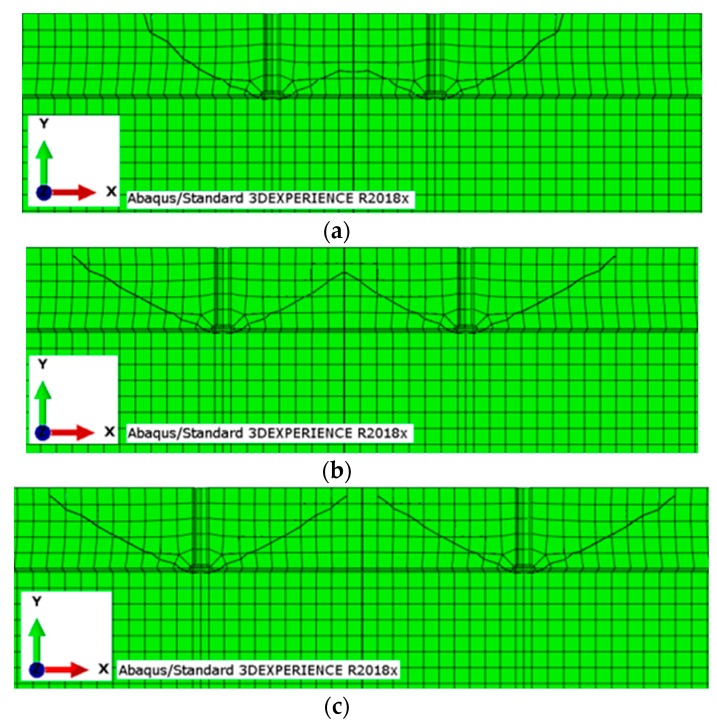

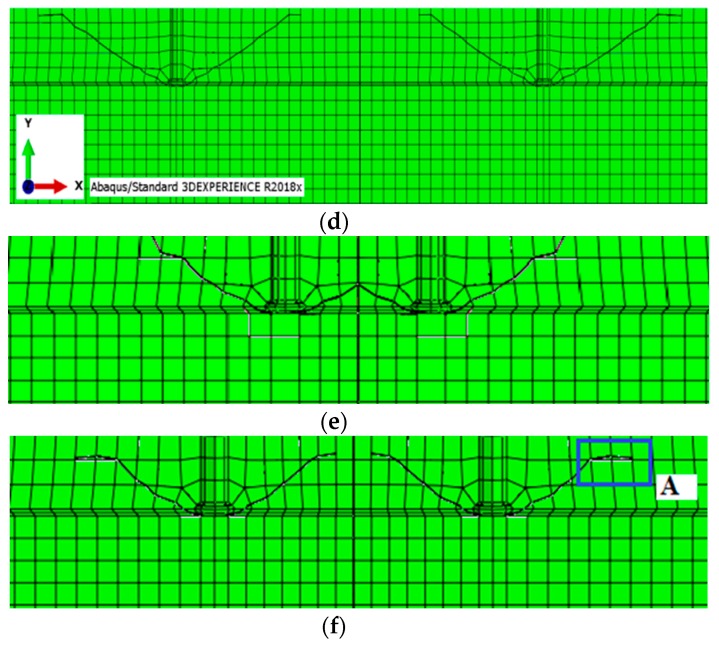
The anchor group effect in cone failure for a group of 2 anchors relative to anchor spacing: (**a**–**d**) *h*_ef_ = 100 mm and the spacing *s* between the holes: (**a**) 100, (**b**) 150, (**c**) 200, (**d**) 300 mm, (**e**,**f**) *h*_ef_ = 50 mm and the spacing *s* between the holes: (**e**) 50 mm, (**f**) 100 mm.

The results from the simulations are illustrated in Figure 6 and Figure 7.

The results from the FEA (Finite Element Analysis) highlight the influence of the group effect for smaller *s*/*h_ef_* ratios, up to 5–5.4. The computed value is significantly higher than that according to the CCD method (<3); however, owing to the inefficiency of the ABAQUS system in precise determination of the fracture trajectory in the final stage of crack propagation (e.g., Figure 7f, detail “A”), the value must be regarded as an estimate.

## 3. Experimental Verification of the FEM Cone Failure Models

**Figure 8:**
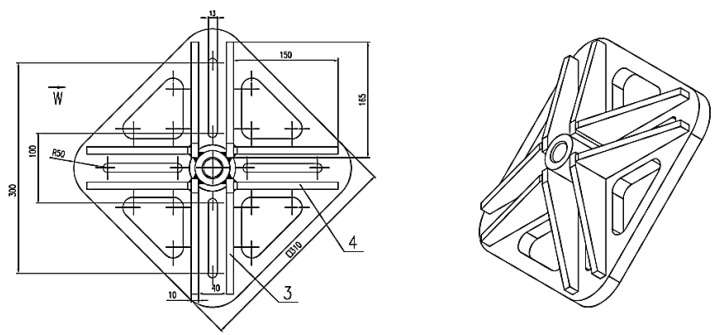
The schematics of the fastening plane for a system of two (four) anchors.

The premise of the experimental part of the study assumed that the group effect in the concrete cone failure of a system of anchors connected using a custom-built fastening plate enables an even distribution of loads on each anchor [25]. Figure 8 shows the structural design of the fastening plate in question built to accommodate two (or four) anchors and to allow the testing of variable anchor spacing settings.

For the sake of accuracy and strength properties, it was vital that the anchors composing the fastening system were of the same stiffness (type, size, and embedment depth). The group effect in a system of anchors subjected to a working load is known to occur under the condition that the axial spacing *s* between the anchors is smaller than the critical spacing (according to the CCD method, for concrete cone failure *s_gr_* = 3*h_ef_*).

**Figure 9:**
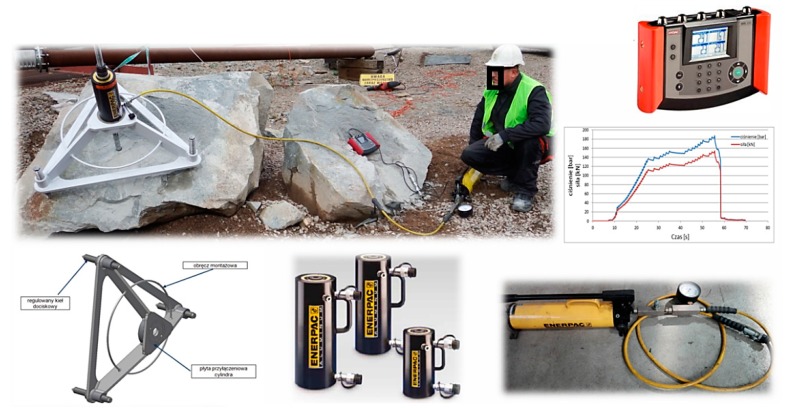
Components of the mobile tensile test setup.

Figure 9 shows the components of the mobile concrete breakout failure test stand: The jig frame for the testing actuator device with three height-adjustable supports, the hydraulic cylinder, the hand pump set with a pressure gauge, and a digital recorder. By calibrating the measurement path, the current force acting on the anchor was determined with high accuracy and precision (the force was calculated from the current pressure in the hydraulic cylinder and the geometrical parameters of the working elements of the cylinder).

**Figure 10:**
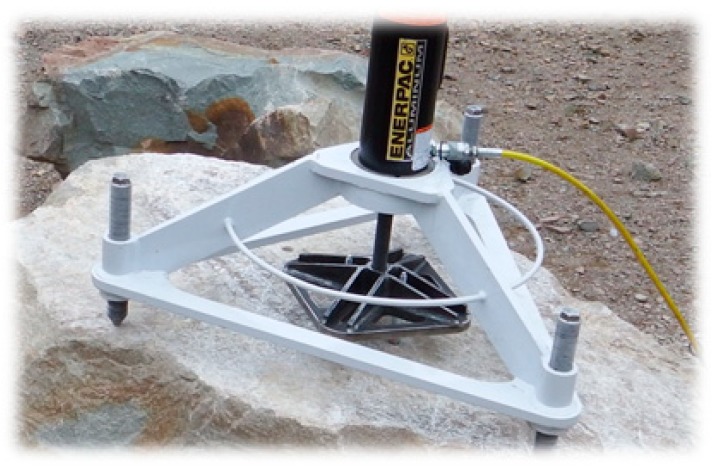
The jig frame for the hydraulic cylinder with the fastening plate and the system of two anchors.

The jig frame with the testing actuator device and the fastening plate for the two- or four-anchor fastener (depending on the tested anchor system variant) are shown in Figure 10.

**Figure 11:**
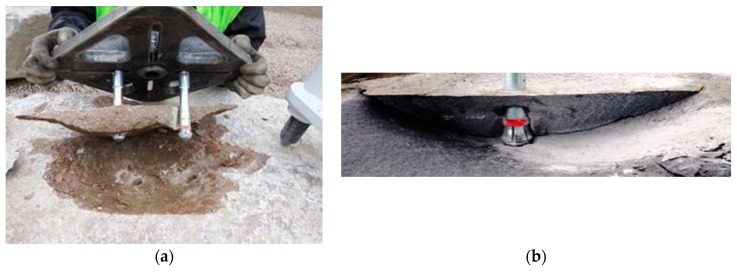
The cone failure specimens obtained in tests for the systems of (**a**) 2 anchors and (**b**) 1 anchor.

The failure cones for the two- and one-anchor systems are presented in Figure 11. In total, several dozen dissimilar specimens were obtained. The scatter of results concerned both the tensile breakout force as well as the size and shape of the breakout cones. The discrepancies can be attributed to the varied internal structure and strength parameters of the rocks subjected to testing. Figure 11a,b provide an illustration of the group effect in the cone failure observed in the two and one-anchor systems.

The failure surface was scanned using a 3D digital laser scanner. The point cloud obtained from the scan was manually processed and converted into an STL triangulated surface. Subsequently, specialist LEIOS 2 R10 software (E.G.S. Srl, 2019, Bologna, Italy,) was used to process and convert the STL model into the *.sat model. The 3D solid model of the failure surface after the breakout failure was subsequently processed with the use of Inventor software to obtain a derivative element in the form of a breakout cone.

**Figure 12:**
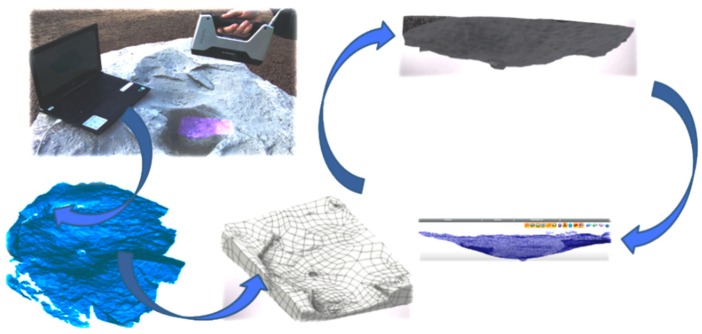
The failure cone modelling procedure.

The data provided by the 3D solid model of breakout cones were used to calculate the failure surface areas, the surface areas of the cone, and the volume of the breakout cone. The failure surface model obtained from 3D scanning enabled us to view crack propagation in any section of a considered specimen; the crack propagation lines obtained in multi-anchor systems are illustrated in Figure 12.

**Figure 13:**
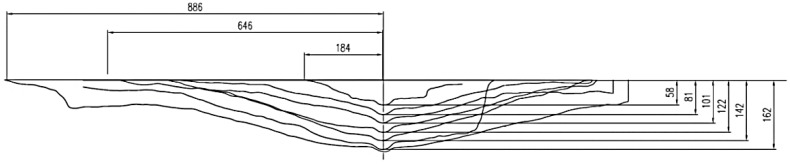
Typical crack propagation for a single-anchor fastener, depending on the effective embedment depth.

Figure 13 shows the typical crack propagation and Figure 12 shows the failure cones of single-anchor fasteners, including crack propagation and length (in the axial section of an anchor) depending on the effective embedment depth.

The figure also displays atypical crack propagation in rock specimens whose structure was cracked or showed other internal defects.

**Figure 14:**
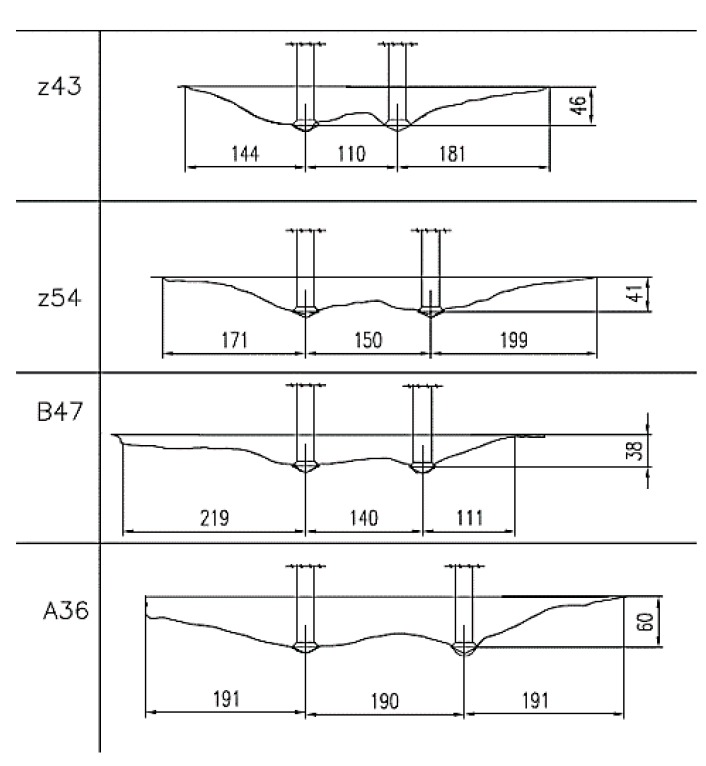
Sections of failure cones for systems of 2 anchors.

The section in Figure 14 runs through the axes of two neighboring anchors.

**Figure 15:**
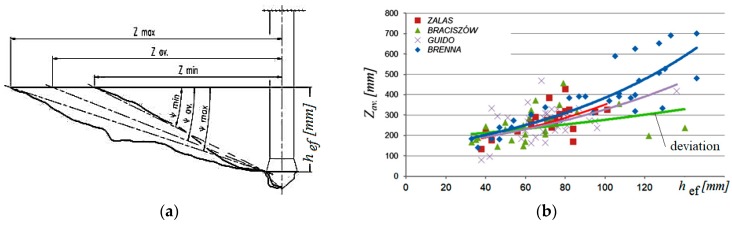
The average breakout cone angle *ψ_av_* (**a**) and crack length *Z_av_* (**b**) as functions of embedment depth *h_ef._*

From the data above, the crack lengths were determined (Figure 15a), measured on the free surface of the base material (corresponding to the average radius of the breakout cone at the base). The pull-out tests were performed on the substrates extracted from four mines: Zalas, Guido, Braciszów, and Brenna [25]. Average breakout cone angles (*ψ_av_*, Figure 15a) and the average crack length (*Z_av_*, Figure 15b) were determined as a function of the effective embedment depth *h_ef_*.

The correlation characteristics observed in the material from Braciszów mine show a deviation from the others. This most probably results from the physical and mechanical parameters of rocks occurring there, i.e., very strong sandstones (*f_c_* = ~155 MPa, *f_t_* = ~8 MPa).

The results from field experiments are summarized in Table 1, which shows that the rocks subjected to testing displayed considerable differences in terms of strength parameters. In addition, they were of a diverse geological structure, including bedding, cracks, and humidity. These factors are likely to have had a major impact on the scatter of the results. Moreover, the stone material showed an exceptionally large angle of internal friction (approx. 49°–53°) compared to the cement material typically described in anchor fasteners, and a much higher resistance to uniaxial compression. The ratio of the average radius of the destruction cone *Z_av_* to the effective embedment depth *h_ef_* (parameter *R*, Table 1) ranged from 3.9 to 4.2. On average, it is almost 2–3 times higher than that in the CCD model. As it results from Figure 13, in uncracked rocks, particularly sandstone, these values are of the order of 5.5 and more. Therefore, for the assumed anchor embedment depths, the possible diameters of the failure cone base could even reach 10*h_ef_*, whereas the CCD value is of the order of 3*h_ef_*, thus exceeding the CCD model recommendations by more than three times.

The obtained results are essential when designing the anchor arrangement or spacing in multi-anchor fasteners to ensure the optimal efficiency of rock-breaking by anchor pull-out.

## 4. Discussion

**Figure 16:**
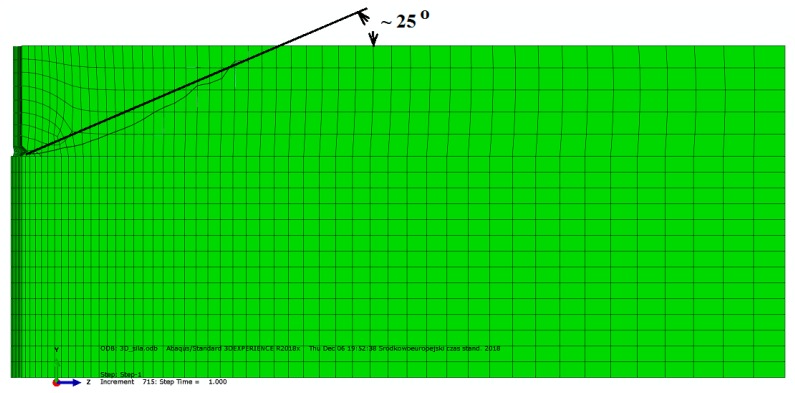
The average breakout cone angle *ψ_av_* (simulations, Figure 7).

The average breakout prism angle obtained from the FEM analyses is significantly lower than that assumed in the CCD model (i.e., ~35°, concrete [10]). In the FEM simulations, the angle is shown to fluctuate around 22° [5] and less (Figure 16). The laboratory tests have shown that for rocks, this angle is significantly smaller (~15°) (see Table 2).

In the experimental tests, it was found that the ratio of mean values of the conical failure radius to the effective embedment depth *h_ef_* ranged from 3.9 to 4.2. This is a value that is, on average, almost 2–3 times higher than assumed in the CCD model (1.5*h_ef_*). For uncracked rocks, especially sandstone, these values are on the order of 5.5 or more.

In the analysis of embedment depths, the diameters of the breakout cone base were shown to reach values of 10*h_ef_*, i.e., more than three times greater than those for use with CCD (3*h_ef_*) instructions. In the case of multiple-anchor applications, the results of FEM analysis indicate a clear effect of the cone fastening for selected proportions *s*/*h_ef_* (*s*—distance between anchor axes). From the simulation, FEM-3D takes into account that for the limit value of the *s*/*h_ef_* ratio, possible interactive reactions of failure are on the order of 5–5.4. This value is much higher than those available with the recommendations of the CCD methods (≤3).

## 5. Conclusions

The purpose of the study was to investigate the usefulness of particular models and calculation methods that are widely employed in the estimation of the breakout strength of anchors in cement/rock base material, i.e., mainly the CCD method. The models allow engineers to determine the critical parameters for the use of undercut anchors in special-purpose rock mining, including extreme conditions, such as methane hazard or geological mining restrictions.

The findings from both the finite element analysis and the experimental tests provide contradicting evidence to the widely accepted models. The dimensions of the failure cones with respect to the tested embedment depths and rock substrates observed in this investigation are far above what results from the standard computational models.

The breakout prism angle *α* that was established using the FEM simulation and the experimental tests is significantly smaller compared to the CCD model. Although the CCD model assumes that the angle in question should be equal to 35°, from the results of the FEM simulations reported in this work, it can be seen that the average angle was 22°. The discrepancies reflect the difference in the simulation conditions, in particular, the embedment depth, rock strength parameters, Poisson’s ratio, or coefficient of sliding friction of the anchor on rock. The stone breakout angle determined in the experimental research was even smaller and fluctuated around 15° or less, depending on the particular bedding or cracking characteristics of the particular rock specimens.

Given the considerable variability in the mining and geological conditions during the experimental stage (including cracking and fracturing of rocks), further studies will need to be undertaken to take these variables into account and increase the precision of the determined parameters.

## Figures and Tables

**Table 1:** Selected test results.

Mine	Rock Type	No. of Tests	Uniaxial Compressive Strength *R_c_*, (MPa)	Uniaxial Tensile Strength *R_rb_*, (MPa)	Cohesive Strength *C,* (MPa)	Angle of Internal Friction *Φ,* (°)	R = *Z_av_*/*h_ef_*	*ψ_av,_*.(°)
ZALAS	porphyry	30	106.5	5.9	8.6	54.0	3.9	15.1
BRACISZÓW	sandstone	27	97.4	6.2	11.9	49.6	4.1	15.3
GUIDO	sandstone	36	155.3	8.0	14.5	49.5	3.9	15.8
BRENNA	sandstone	22	58.8	3.9	6.0	53.0	4.2	13.7
Medium							4.03	14.96

**Table 2:** Comparison of the values of the tested parameters *R* and *ψ_av._*

-	CCD-Concrete	FEM in Literature	Analytical-Concrete	FEM ABAQUS—Own Research—Rocks	Laboratory Test-Rocks
*R* (medium)	1.5 [26]		~2 [8,23]	2.5–3	~4
*s_gr_* (Figure 4)	3		~4 [8,14,23]	~5	~10
*ψ_av._*(medium)	35° [26]	22° [5,16,26]	37.5° [13]	25° (Figure 16)	~15°
			22°–25° [8]		
			27° [23]		
